# *Taenia martis* Neurocysticercosis-Like Lesion in Child, Associated with Local Source, the Netherlands

**DOI:** 10.3201/eid3003.231402

**Published:** 2024-03

**Authors:** Hendriekje Eggink, Miriam Maas, Judith M.A. van den Brand, Jasja Dekker, Frits Franssen, Eelco W. Hoving, Laetitia M. Kortbeek, Mariëtte E.G. Kranendonk, Linda C. Meiners, Anne E. Rittscher, Jeroen Roelfsema, Elisabeth H. Schölvinck

**Affiliations:** University Medical Center Groningen, Groningen, the Netherlands (H. Eggink, L.C. Meiners, E.H. Schölvinck);; National Institute for Public Health and the Environment, Bilthoven, the Netherlands (M. Maas, F. Franssen, L.M. Kortbeek, J. Roelfsema);; Utrecht University, Utrecht, the Netherlands (J.M.A. van den Brand, A.E. Rittscher);; Jasja Dekker Dierecologie B.V., Arnhem, the Netherlands (J. Dekker);; Princess Maxima Center, Utrecht (E.W. Hoving, M.E.G. Kranendonk)

**Keywords:** Neurocysticercosis, Mustelidae, Taenia, Cestoda, zoonoses, parasites, the Netherlands

## Abstract

A neurocysticercosis-like lesion in an 11-year-old boy in the Netherlands was determined to be caused by the zoonotic *Taenia martis* tapeworm. Subsequent testing revealed that 15% of wild martens tested in that region were infected with *T. martis* tapeworms with 100% genetic similarity; thus, the infection source was most likely local.

The zoonotic *Taenia martis* tapeworm lives in mustelid intestines and has been reported across Europe (*1*). Human infection is thought to occur by accidental ingestion of eggs in mustelid feces and can lead to cysticercosis-like lesions, reported for only 6 adults in France, Germany, and Switzerland (*2*–*7*). We report a *T. martis* neurocysticercosis-like lesion in a child in the Netherlands.

## The Study

In 2020, an 11-year-old boy was referred to the emergency department of University Medical Center Groningen (Gronigen, the Netherlands). Three days earlier, he had awakened with a frontal headache that intensified within 1 hour and led to nausea and vomiting. Symptoms resolved after sleep. On the evening of his referral to the emergency department, the boy suddenly became nauseous and pale, unable to speak, and in a decreased state of consciousness. His altered mental status continued for 20 minutes and his speech arrest for 90 minutes; a headache followed. No urine incontinence or tongue bite were noted. His medical history revealed only allergic rhinitis. He was an enthusiastic runner in the northern Netherlands woods and spent holidays in different nature areas of western Europe.

**Figure 1:**
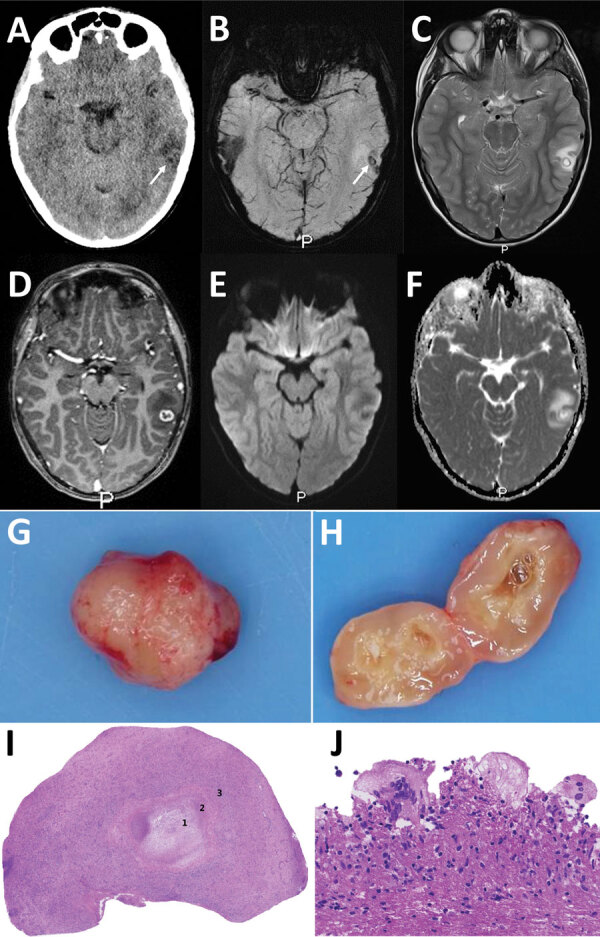
Diagnostic imaging of the brain and cystic lesion resected from boy with neurocysticercosis-like lesion, the Netherlands. A) Axial computed tomography showing edema in the left temporal lobe with a barely visible hypointense round lesion with a noncalcified, isointense rim (arrow). B–F) Axial magnetic resonance images at slightly different levels through the cystic lesion with surrounding edema in the left temporal lobe, showing a hypointense ring on susceptibility-weighted image (B) using minimum intensity projection (arrow) and on T2-weighted image (C), suggestive of a fibrotic capsule. D) Three-dimensional T1-weighted image showing a slightly irregular enhancement of the rim. E, F) On diffusion-weighted image (E) and apparent diffusion coefficient map (F), the rim is isointense and central diffusion restriction is absent, excluding a bacterial abscess. G, H) Macroscopic picture of the lesion showing a round nodule (G) and a cyst-like lesion (H) on cut section with a white-greyish central area surrounded by a thin capsule. I, J) Microscopic images showing a necrotic core (1) surrounded by a rim of fibrosis (2) and a mixed inflammatory response (3) (I) and multinuclear foreign-body-type giant cells (J).

At the emergency department, his symptoms had resolved, and initial examination revealed no neurologic or laboratory test abnormalities. Computed tomography of his brain without contrast showed a hypodense area in the left temporal lobe and a barely discernable ringlike lesion with an isointense rim, without calcification (Figure 1, panel A). A cerebral venous sinus thrombosis was excluded. Magnetic resonance imaging (MRI) revealed a 13-mm round lesion with edema in the dorsal left temporal lobe and a hypointense rim on susceptibility-weighted and T2-weighted images (Figure 1, panels B, C), suggesting a fibrotic capsule, enhanced on 3-dimensional T1-weighted images (Figure 1, panel D). On diffusion-weighted images, no central diffusion restriction was seen (Figure 1, panels E, F). Initially, a brain tumor of undefined origin was proposed, but a second viewing suggested neurocysticercosis. Results of serologic testing of 2 samples collected 3 weeks apart, tested for *T. solium* tapeworms via a Centers for Disease Control and Prevention immunoblot recombinant antigen (rT24H antigen and LLGP) (*8*,*9*), were, however, negative.

The boy remained symptom free and because of the differential diagnosis of a brain tumor was referred to the national center for pediatric oncology in the Netherlands, the Princess Maxima Center (Utrecht, the Netherlands). Two weeks after the initial visit to University Medical Center Groningen, the patient underwent an uncomplicated craniotomy, and a cyst was extirpated in toto (Figure 1, panel G). Macroscopically, the lesion appeared to be an intact cystic round nodule on cut section with a white-greyish central area surrounded by a thin capsule (Figure 1, panel H). Microscopic examination revealed a necrotic core surrounded by fibrin and fibrosis (Figure 1, panel I) with adjacent multinuclear foreign body–type giant cells and an inflammatory infiltrate including plasma cells and eosinophilic neutrophils (Figure 1, panel J). No tegument or calcareous corpuscles were seen. After ruling out common pathogenic microorganisms, we determined that those features could fit well with the second (necrotic) stage of neurocysticercosis (*10*).

PCR analysis of the cyst material was performed by using the 12S rRNA gene as target (*11*) (primers: forward 5′-AAAIGGTTTGGCAGTGAGIGA-3′; reverse 5′GCGGTGTGTACITGAGITAAAC-3′) and with *T. saginata* DNA as positive control. PCR revealed a tapeworm infection, and sequencing indicated *T. martis* (Appendix). Those findings led to the final diagnosis of a stage 2 neurocysicercosis-like lesion, based on the *T. martis* infection. The patient received albendazole (2×/d for 1 week). Follow-up MRI of the brain 1.5 months after surgery showed only the resection cavity, and the boy has remained symptom free.

To explore potential sources, we investigated stone martens (*Martes foina*) that had been killed as part of ongoing predator control in 2020 and 2021 in Friesland, a northern province of the Netherlands. We checked their intestines macroscopically for *T. martis* tapeworms and collected intestinal content from multiple parts of the intestine to submit for molecular detection of *T. martis* tapeworm DNA. We extracted DNA from collected tapeworms and all intestinal scrapings by using the DNeasy Blood and Tissue Kit (QIAGEN, https://www.qiagen.com). We also performed conventional PCR targeting the *CO1* gene on the patient material, using primers previously reported (*12*), with slight modification of the primers (forward, 5′-TTTTTTGGGCATCCTGAGGTTTAT-3′; reverse, 5′-TAACGACATAACATAATGAAAATG-3′), followed by electrophoresis using 1.8% agarose gel PCR. We sequenced samples with bands matching the positive control, obtained from a *T. martis* worm collected at the start of the project, by using BaseClear (Leiden, https://www.baseclear.com) and performed BLAST analysis (https://www.ncbi.nlm.nih.gov/blast/Blast.cgi). 

**Figure 2:**
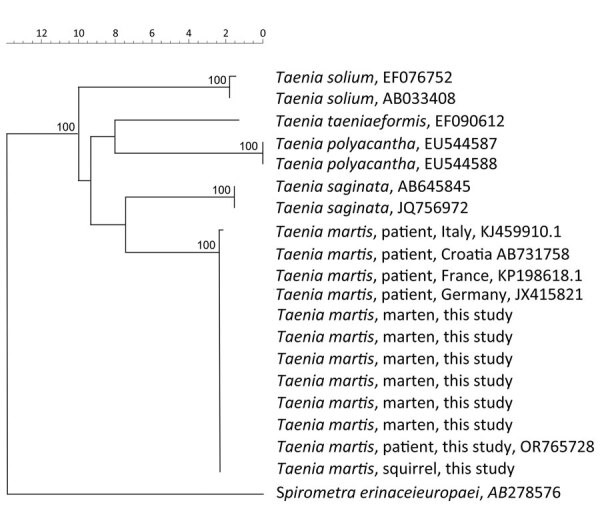
Phylogenetic analysis of the partial *CO1* gene of *Taenia martis* tapeworm samples from a patient, martens, and a squirrel in the Netherlands and reference sequences. GenBank accession numbers are shown when available. The tree is based on multiple alignment with Jukes and Cantor correction and neighbor-joining cluster analysis. Branch quality was determined by bootstrap analysis with 10,000 simulations. Reference sequences were from patients from Italy (GenBank accession no. KJ459910.1), Croatia (accession no. AB731758), France (accession no. KP198618.1), and Germany (accession no. JX415821). Moreover, *T. saginata* (accession nos. AB645845 and JQ756972), *T. solium* (accession nos. EF0767752 and AB033408), *T. polyacantha* (accession nos. EU544587 and EU544588), and *T. taeniaeformis* (accession no. EF090612) were included in the phylogenetic analysis. The cestode *Spirometra erinaceieuropaei* (accession no. AB278576) was included as outgroup. Scale bar indicates nucleotide substitutions/site.

Of the 214 collected stone martens, sequences of 32 (15%, 95% CI 10%–20%) intestinal scraping samples matched *T. martis* sequences from GenBank, including samples from 7 stone martens in which adult tapeworms were macroscopically detected and confirmed by PCR and sequencing to be *T. martis*. Genetic analysis showed 100% similarity between the *T. martis* sequence of the patient (GenBank accession no. OR765728) and those from the martens. In addition, a *T. martis* sequence from larval cestode from a squirrel collected in 2014 in the Netherlands (provided by Herman Cremers) was 100% identical to the sequence from the patient. Sequences from *T. martis* tapeworms collected in Switzerland, Croatia, France, and Germany were 100% identical and from Italy 99.6% identical to the sequences of the martens from the Netherlands (Figure 2).

## Conclusions

To our knowledge, human *T. martis* cysticercosis has been reported for only 6 adults. Two cases involved a *T. martis* neurocysticercosis-like lesion (*2*,*7*), and the others involved the eye, peritoneum, and pouch of Douglas (*3*–*6*). All 6 patients were immunocompetent women: 5 tended and ate from vegetable gardens, 5 lived in rural areas, and 3 were frequent hikers/dog owners. The boy we report also spent a lot of time in the forest. 

Stone martens are synanthropic mustelids and will eat fruit or scavenge scraps from compost heaps in gardens and barnyards. It is hypothesized that consuming contaminated vegetables or fruit or accidentally ingesting *T. martis* eggs after contact with contaminated soil may lead to (neuro)cysticercosis-like infection caused by *T. martis* tapeworms.

Neurocysticercosis involves infection of the central nervous system by the larval stage of the pork tapeworm *T. solium* (*13*). The MRI features for the boy with a *T. martis* neurocysticercosis-like lesion and the patient in France resemble those caused by *T. solium* tapeworms (*2*). Features depend on stage of the infection (*14*). No specific serologic test is available for *T. martis* infection, and the extent of cross-reactivity between *T. solium* and *T. martis* antibodies in available serology tests is unknown. Serologic test results for the 6 adult patients showed mixed signals, including positive signals against *Echinococcus multilocularis* crude larval antigen extract (that could not be repeated in confirmatory assays) (*5*) and *T. solium* (*2*,*5*), although others have reported negative serologic test results for those parasites (*3*,*4*). Confirming the diagnosis requires detecting parasite DNA by PCR and sequencing to differentiate between *Taenia* species. The availability of differentiating molecular methods may have resulted in increased diagnoses of *T. martis* infections, possibly previously misdiagnosed as *T. solium* infections (*3*).

The finding of *T. martis* tapeworms in the patient and the stone martens we investigated from the northern part of the Netherlands strongly suggest a local source of infection. Although the prevalence of *T. martis* tapeworms can vary widely regionally (*15*), studies in host and reservoir species suggest widespread appearance of *T. martis* tapeworm in mustelids in Europe (*1*), and underrecognition and underreporting of cysticercosis caused by infection with this tapeworm is probable.

Appendix Alignment of the 12S sequence of cyst material from *Taenia martis* neurocysticercosis-like lesion.
