# Supplementary material for: Taenia martis Neurocysticercosis-Like Lesion in Child, Associated with Local Source, the Netherlands
Source: Emerg Infect Dis. 2024 Mar;30(3):555–9. doi: 10.3201/eid3003.231402 (PMC10902551; doi:10.3201/eid3003.231402)
Supplement: Appendix — Alignment of the 12S sequence of cyst material from Taenia martis neurocysticercosis-like lesion. [file 23-1402-Techapp-s1.pdf]

Article DOI: https://doi.org/10.3201/eid3003.231402

EID cannot ensure accessibility for supplementary materials supplied by authors. Readers who have difficulty accessing supplementary content should contact the authors for assistance.

Taenia martis Neurocysticercosis-Like Lesion in Child, Associated with Local Source, the Netherlands

Appendix

Alignment of the 12S sequence

Shown below is the alignment of the 12S sequence from the PCR product derived from the patient with 12S sequences with other tapeworm species. Similar nucleotides are indicated by a dot. Reference sequences are: *T. martis* KT943414, *T. crassiceps* NC_002547, *H. taeniaeformis* EU219548, *T. saginata* KC344689 and *T. solium* AB086256.

```
Sample          ttctttttaggggaaggtgtggtgtaaaggatgttccgcctattatttta
T.martis        ..................................................
T.crassiceps    ..................................................
H.taeniaeformis .................................a........a..a....
T.saginata      .............................................a....
T.solium        ....................a........................a....

Sample          cttttattatgttggtgtatatctgatttaatattattgcttaatggttt
T.martis        ..................................................
T.crassiceps    .................................................a
H.taeniaeformis ..........a..............g...............g.....cag
T.saginata      .........................g...........c.t.g.g..a.g.
T.solium        .........................g...g.........t.g...a..a.

Sample          --aagtttgtgta-tatttatttaagtcaagtctatgtgctgcttataaa
T.martis        ..................................................
T.crassiceps    ..................................................
H.taeniaeformis ..-.ag........gtg.........c...............t.......
T.saginata      at...........ggt....g.....ct......................
T.solium        ..-.a.........gtg...g.....c.......................

Sample          agtgttcatgcgttactttaataatatgttggttgaaa-tatta--atta
T.martis        ..................................................
T.crassiceps    ...a..........................a.a................g
T.taeniaeformis ...a............a.......gtat..aa...t..........ga.g
```

```
T.saginata       ...a.a..........a..g....ag.t..a....t..g.gc....t.at
T.solium         ...a..t............g....ag.t..a....t.....c..tt..at

Sample           atttaggacttaatagtaatatttaattaatttgtagatgtgaatata
T.martis         ................................................
T.crassiceps     ....................g........g......a...........
H.taeniaeformis  .............a......g...........a...a.......ata.
T.saginata       ...c.........a.....cg..a.....g.....ta.......gta.
T.solium         .............g........ca.....g.....t.g......ata.
```
